# *Low Temperature-Induced 30* (*LTI30*) positively regulates drought stress resistance in *Arabidopsis*: effect on abscisic acid sensitivity and hydrogen peroxide accumulation

**DOI:** 10.3389/fpls.2015.00893

**Published:** 2015-10-20

**Authors:** Haitao Shi, Yinhua Chen, Yongqiang Qian, Zhulong Chan

**Affiliations:** ^1^Hainan Key Laboratory for Sustainable Utilization of Tropical Bioresources, College of Agriculture, Hainan UniversityHaikou, China; ^2^State Key Laboratory of Tree Genetics and Breeding, Research Institute of Forestry, Chinese Academy of ForestryBeijing, China; ^3^Key Laboratory of Plant Germplasm Enhancement and Specialty Agriculture, Wuhan Botanical Garden, Chinese Academy of SciencesWuhan, China

**Keywords:** *Low Temperature-Induced 30*, abscisic acid, hydrogen peroxide, drought stress, reactive oxygen species, *Arabidopsis*

## Abstract

As a dehydrin belonging to group II late embryogenesis abundant protein (LEA) family, *Arabidopsis Low Temperature-Induced 30 (LTI30)/XERO2* has been shown to be involved in plant freezing stress resistance. However, the other roles of *AtLTI30* remain unknown. In this study, we found that the expression of *AtLTI30* was largely induced by drought stress and abscisic acid (ABA) treatments. Thereafter, *AtLTI30* knockout mutants and overexpressing plants were isolated to investigate the possible involvement of *AtLTI30* in ABA and drought stress responses. *AtLTI30* knockout mutants were less sensitive to ABA-mediated seed germination, while *AtLTI30* overexpressing plants were more sensitive to ABA compared with wild type (WT). Consistently, the *AtLTI30* knockout mutants displayed decreased drought stress resistance, while the *AtLTI30* overexpressing plants showed improved drought stress resistance compared with WT, as evidenced by a higher survival rate and lower leaf water loss than WT after drought stress. Moreover, manipulation of *AtLTI30* expression positively regulated the activities of catalases (CATs) and endogenous proline content, as a result, negatively regulated drought stress-triggered hydrogen peroxide (H_2_O_2_) accumulation. All these results indicate that *AtLTI30* is a positive regulator of plant drought stress resistance, partially through the modulation of ABA sensitivity, H_2_O_2_ and proline accumulation.

## Introduction

Plants are exposed to various environmental conditions, however, plants can not change their location to avoid unfavorable circumstance (Shi et al., 2013a,b, 2014a,b). Among multiple stresses, drought stress is one of the most harsh environmental stresses (Seki et al., 2007; Harb et al., 2010; Chan and Shi, 2015). To date, plants have developed sophisticated strategies to counteract sudden environmental changes. Many secondary messengers, including abscisic acid (ABA), and hydrogen peroxide (H_2_O_2_), are involved in plant stress transduction (Seki et al., 2007; Yu et al., 2008; Fujii et al., 2009; Cutler et al., 2010; Qin et al., 2011). Both endogenous concentrations and the underlying signaling pathways of ABA and H_2_O_2_ play essential roles in plant drought stress responses (Fujii et al., 2009; Cutler et al., 2010; Harb et al., 2010; Qin et al., 2011; Munemasa et al., 2013).

*Arabidopsis Low Temperature-Induced 30* (LTI30)/XERO2 belongs to the group II late embryogenes abundant protein (LEA)/dehydrin family (Rouse et al., 1996). The transcript level of *AtLTI30/XERO2* can be induced by ABA, cold, dehydration, wounding, and salt stresses (Welin et al., 1994; Rouse et al., 1996; Nylander et al., 2001; Chung and Parish, 2008). Overexpression of *AtLTI30/XERO2* enhances freezing stress resistance in *Arabidopsis* (Puhakainen et al., 2004). AtCBF1, AtCBF2, and AtCBF3 (also known as AtDREB1b, AtDREB1c, and AtDREB1a, respectively) are important transcription factors in plant abiotic stress responses. To date, many stress-responsive genes with C-repeat (CRT)/dehydration-responsive element (DRE) in the promoters have been identified as the direct targets of AtCBFs. These genes include *COR* (*cold regulated*), *ERD* (*early responsive to dehydration*), *KIN* (*cold inducible*), *LTI* (*low-temperature induced*), and *RD* (*responsive to dehydration*) (Gilmour et al., 1998; Zarka et al., 2003; Cook et al., 2004; Thomashow, 2010). Using multiple combinations of mutations in the promoter of *AtLTI30/XERO2*, Chung and Parish (2008) found that two of the ACGT and DRE/CRT elements in the promoter of *AtLTI30/XERO2* were essential for cold and ABA transcriptional induction of *AtLTI30/XERO2*. Mouillon et al. (2006) found that the lysine-rich segment of AtLTI30/XERO2 showed sequence similarity with the animal chaperone heat shock protein 90 (HSP90). The conserved segments of AtLTI30 exerted its biological function more locally upon interaction with specific biological targets. Moreover, Eriksson et al. (2011) identified three factors that regulate the lipid interaction of LTI30 *in vitro*, including the pH dependent His on/off switch, reversal of membrane binding by proteolytic digestion, and phosphorylation by protein kinase C.

Although *AtLTI30* has been shown to be involved in plant freezing stress resistance, the other roles of *AtLTI30* and the underlying mechanisms remain unknown. In this study, the expression and function of *AtLTI30* were characterized in response to drought stress treatment. We investigated the effects of manipulation of *AtLTI30* expression on drought stress resistance, as well as the underlying mechanisms. The results indicate that *AtLTI30* is a positive regulator of drought stress resistance in *Arabidopsis*.

## Materials and Methods

### Plant Materials and Growth Conditions

After stratification at 4°C for 3 days in darkness, *Arabidopsis thaliana* seeds were sown in soil in a growth chamber, and watered with a nutrient solution twice per week. The growth chamber was controlled at 23°C, with an irradiance of about 150 μmol quanta m^-2^ s^-1^, under 65% relative humidity and 16-h light and 8-h dark cycles. The *lti30-1* (SALK_114915) and *lti30-2* (SALK_016819) mutants were obtained from the *Arabidopsis* Biological Resource Center (ABRC).

### RNA Isolation, Semi-quantitative RT-PCR and Quantitative Real-time PCR

Total RNA was extracted and purified using TRIzol reagent (Invitrogen, Carlsbad, CA, USA) and RQ1 RNase-free DNase (Promega, Madison, WI, USA). First-strand cDNA was synthesized from total RNA using reverse transcriptase (TOYOBO, Osaka city, Japan) as (Shi et al., 2013a,b, 2014a,b, 2015) previously described. Semi-quantitative RT-PCR was performed as Shi et al. (2013a) described using *ubiquitin 10* (*UBQ10*) as the internal control. Quantitative real-time PCR was performed using the CFX96^TM^ Real-Time System (BIO-RAD, Hercules, CA, USA) and the comparative ΔΔCT method with *UBQ10* as a reference gene following (Shi et al., 2013a,b, 2014a,b, 2015). The primers of *UBQ10* (At4g05320) and *LTI30* (At3g50970) are shown in Supplementary Table S1.

### Construction of Vectors and Generation of Transgenic Lines

For the *pLTI30::β-glucuronidase* (*GUS*) transgenic construction, the promoter region of *AtLTI30* was amplified by PCR and inserted into the *BamHI* site of the pBI101.2 vector with kanamycin resistance. For *AtLTI30* overexpressing transgenic construction, the coding region of *AtLTI30* was amplified by PCR and inserted into the *SmaI/XhoI* sites of the pBIM vector with kanamycin resistance under the control of the cauliflower mosaic virus (CaMV) 35S promoter (Yang et al., 2005). The responsible primers for the above vector constructions are shown in Supplementary Table S2. The recombinant constructions were confirmed by DNA sequencing and introduced into wild type (WT) plants of Columbia-0 (Col-0) using *Agrobacterium tumefaciens* strain GV3101-mediated transformation and the floral dip method (Clough and Bent, 1998). Thereafter the transgenic plants were selected on MS medium using kanamycin resistance, and further confirmed by PCR analysis.

### GUS Staining and Quantification of GUS Activity

Glucuronidase staining and quantification of GUS activity were performed as Jefferson et al. (1987) previously described. For GUS staining, *proLTI30::GUS* transgenic plants were incubated in staining solution (100 mM sodium phosphate buffer, pH 7.5, 0.5 mM K_3_[Fe(CN)_6_], and 0.5 mM K_4_[Fe(CN)_6_], 10 mM EDTA, 1.0 mM 5-bromo–chloro-3-indolyl-β-*D*-glucuronide and 0.1% Triton X-100) at 37°C for 8 h, after which the plants were incubated in 70% ethanol to remove chlorophyll. GUS activity was quantified by detecting the conversion of 4-methylumbelliferone from the substrate 4-methylumbelliferyl-β-glucuronide in the same concentration of protein extract, as Jefferson et al. (1987) previously described.

### Determination of ABA Sensitivity

For ABA sensitivity assay, different genotypes of *Arabidopsis* seeds were sterilized with 70% (v/v) ethyl alcohol, 5% (w/v) NaClO, and deionized water. The seeds were stratified at 4°C for 3 days, thereafter were sown on Murashige and Skoog (MS) medium plates containing different concentrations of ABA. Germination ratios as seen with emerged radicals and green cotyledons were scored after 10 days in the growth chamber. Stomatal aperture in *Arabidopsis* leaves was determined as Shi et al. (2013a) described.

### Plant Drought Stress Treatment and Drought Stress Resistance Assay

For drought stress, 14-day-old *Arabidopsis* plants in the soil were subjected to control (well-watered) and drought stress (withheld water) conditions for another 21 days. More than three pots of each variety (27 plants) were used in each biological repeat, and all these pots with plants were rotated daily during drought stress to minimize the environment effect. The survival rate of stressed plants was recorded after re-watered for 4 days later than 21 days of drought stress treatment. Relative *in vitro* leaf water loss rate was expressed as percent change in leaf fresh weight (FW) as (Shi et al., 2013a,b, 2014a,b, 2015) described.

### Quantification of Hydrogen Peroxide (H_2_O_2_) and Catalase (CAT) Activity

The concentration of H_2_O_2_ in plant leaves was determined using the titanium sulfate method, and CAT (EC 1.11.1.6) activity was determined using the enzyme assay kit as (Shi et al., 2013a,b, 2014a,b, 2015) previously reported.

### The Determination of Proline Content

Quantification of endogenous proline content was performed as Shi et al. (2013a,b) previously described. Briefly, endogenous proline in plant leaves was extracted using 3% (w/v) sulfosalicylic acid, and the red solution at the absorbance of 520 nm was determined by adding the mixtures of ninhydrin reagent and glacial acetic acid to the extractions.

### Statistical Analysis

All experiments were performed with at least three biological repeats, and plant leave samples in each biological repeat were mixture samples of at least 10 plants per genotype. Student’s *t*-test and analysis of variance (AVOVA) were used to analysis the significant difference, and asterisk symbols (^∗^) indicate the significant differences of *p* < 0.05 in comparison to WT.

## Results

### The Expression Pattern of *AtLTI30*

**FIGURE 1:**
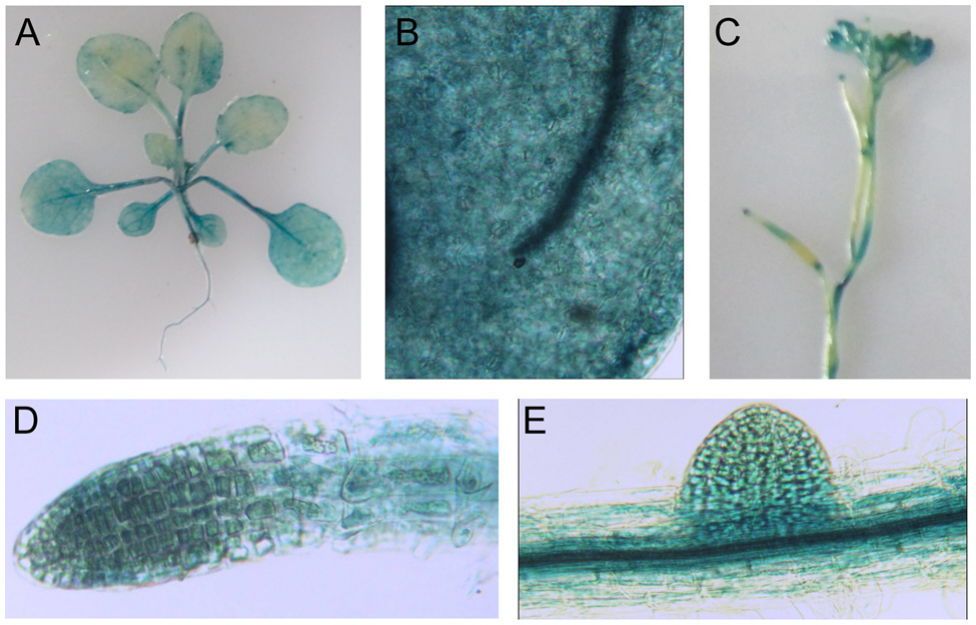
**Glucuronidase staining of *proLTI30::GUS* transgenic plants in different organs. (A)** GUS staining of 21-day-old of *proLTI30::GUS* transgenic plants. **(B–E)** GUS staining of *proLTI30::GUS* transgenic plants in leaves **(B)**, flowers **(C)**, primary root **(D)**, and lateral root **(E)**.

**FIGURE 2:**
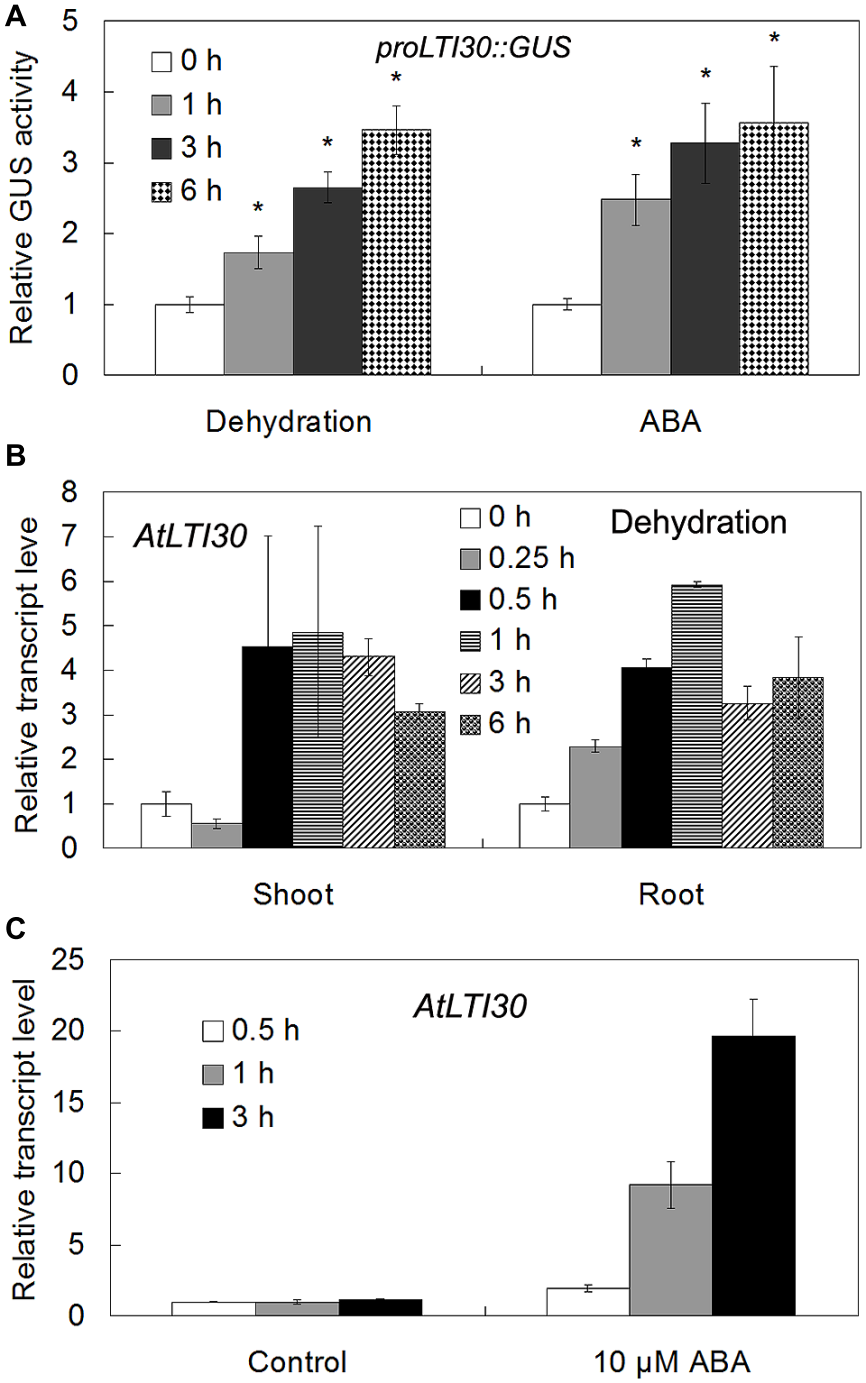
**The expression levels of *AtLTI30* in response to different stress treatments. (A)** The GUS activities of *proLTI30::GUS* transgenic plants in response to different stress treatments. For the assay, 14-day-old of *proLTI30::GUS* seedlings were subjected to dehydration (un-covering the plate in the growth chamber), or transferred to fresh MS liquid medium containing 50 μM ABA, and samples were collected at 0, 1, 3, and 6 h after treatments. The GUS activity of *proLTI30::GUS* at 0 h of treatment was normalized as 1.0. The results shown are the means ± SDs of three biological repeats, and asterisk symbols (^∗^) indicate the significant difference of *p* < 0.05 compared to the expression at 0 h of treatment. **(B,C)** The transcript levels of *AtLTI30* in response to dehydration **(B)** and ABA **(C)** using the publicly available microarray data (http://bar.utoronto.ca/efp/cgi-bin/efpWeb.cgi).

Using *proLTI30::GUS* transgenic plants, we found that *AtLTI30* was widely expressed in leaves, stems, flowers, primary roots and lateral roots (**Figures 1A–E**). Moreover, we found that the GUS activities of *proLTI30::GUS* transgenic plants were significantly increased after dehydration stress and ABA treatments for 1, 3, and 6 h through GUS activity assay (**Figure 2A**). The GUS result is consistent with previous studies that ABA and dehydration induced the transcript level of *AtLTI30* using northern blot (Welin et al., 1994; Rouse et al., 1996; Nylander et al., 2001; Chung and Parish, 2008). Moreover, we also found that the transcript levels of *AtLTI30* are induced by both dehydration and ABA treatments (**Figures 2B,C**) using the publicly available microarray data (http://bar.utoronto.ca/efp/cgi-bin/efpWeb.cgi) (Winter et al., 2007). These results indicate the possible link between *AtLTI30* and these stress treatments, and suggest the possible involvement of *AtLTI30* in drought stress responses in *Arabidopsis*.

### Isolation of *AtLTI30* Knockout Mutants and Overexpressing Plants

**FIGURE 3:**
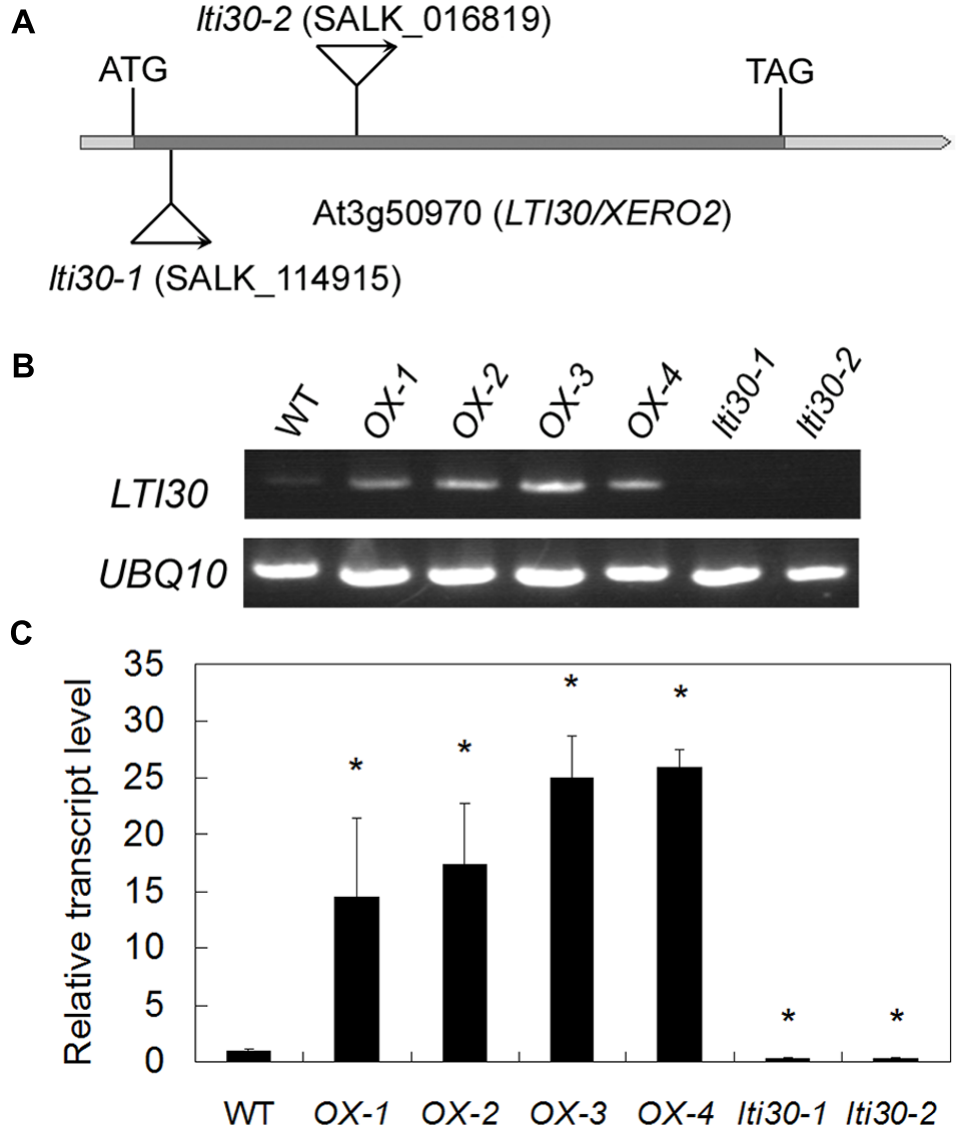
**Characterization of *lti30* knockout mutants and *AtLTI30* overexpressing plants. (A)** Schematic diagrams of the T-DNA position of *lti30-1* and *lti30-2* mutants. **(B,C)** The transcript level of *AtLTI30* in *lti30* knockout mutants and *AtLTI30* overexpressing plants assayed by semi-quantitative RT-PCR **(B)** and quantitative real-time PCR **(C)**. The expression of *UBQ10* was used as an internal control. The results shown are the means ± SDs of three biological repeats, and asterisk symbols (^∗^) indicate the significant difference of *p* < 0.05 in comparison to wild type (WT).

To further reveal the *in vivo* role of *AtLTI30*, we isolated the T-DNA mutants with T-DNA insertion in the extron of *AtLTI30* [*lti30-1* (SALK_114915) and *lti30-2* (SALK_016819)] (**Figures 3A–C**), and constructed *AtLTI30* overexpressing transgenic plants (**Figures 3B,C**). Using semi-quantitative RT-PCR analysis, the transcript of *AtLTI30* was un-detectable in *lti30-1* and *lti30-2* mutants (**Figure 3B**). Consistently, quantitative real-time PCR analysis also showed that the transcript level of *AtLTI30* was largely inhibited in *lti30-1* and *lti30-2* mutants, with about 30% of *AtLTI30* transcripts compared with WT plants (**Figure 3C**). Moreover, the *AtLTI30* overexpressing transgenic plants displayed significantly higher *AtLTI30* transcripts than WT plants, with 15–25-folds higher *AtLTI30* transcripts compared with WT, and the homozygous transgenic plants (OX-2 and OX-3) were chosen for further analysis (**Figures 3B,C**).

### *AtLTI30* Positively Regulates Plant Sensitivity to ABA

**FIGURE 4:**
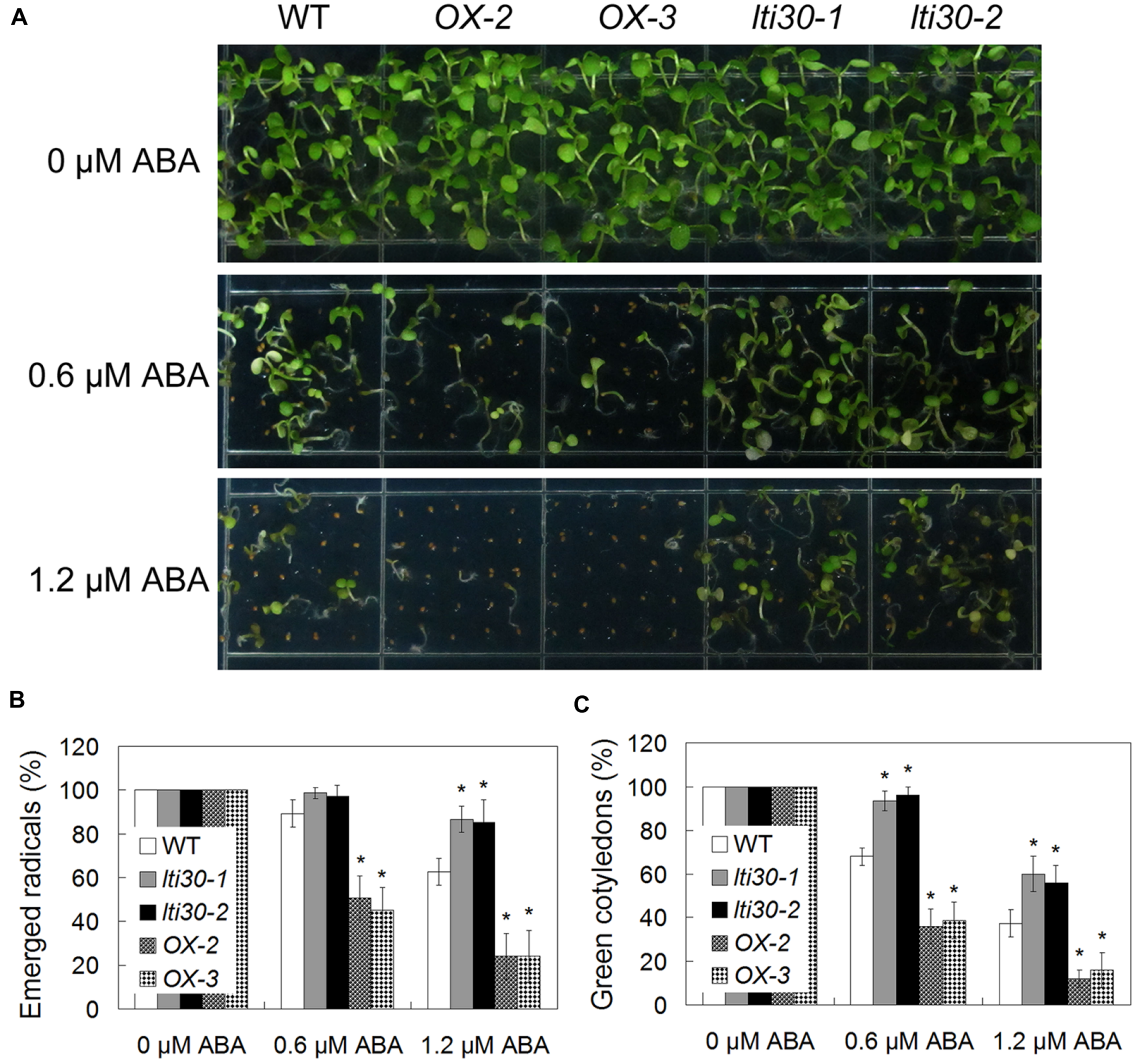
**Modulation of *AtLTI30* expression positively affects plant sensitivity to ABA. (A)** The photograph showing 10-day-old of *Arabidopsis* plant seedlings after grown on MS plate containing different concentrations of ABA. **(B,C)** Germination rates of plant seeds on MS plate containing different concentrations of ABA that were determined as emerged radicals **(B)** and green cotyledons **(C)**. The results shown are the means ± SDs of three biological repeats, and asterisk symbols (^∗^) indicate the significant difference of *p* < 0.05 in comparison to WT.

Since the expression of *AtLTI30* was increased after ABA treatment, the responses of the WT, *lti30* knockout mutants, and *AtLTI30* overexpressing plants to ABA were further compared. Germination of *AtLTI30* overexpressing plant seeds was severely inhibited after ABA treatment, as shown with less emerged radical, less green cotyledon compared with those of WT (**Figures 4A–C**). On the contrary, *lti30-1* and *lti30-2* mutants showed more emerged radicals and more green cotyledons than those of WT (**Figures 4A–C**). These results indicate that modulation of *AtLTI30* expression positively affects ABA sensitivity in seed germination stage.

### *AtLTI30* Positively Regulates Drought Stress Resistance

**FIGURE 5:**
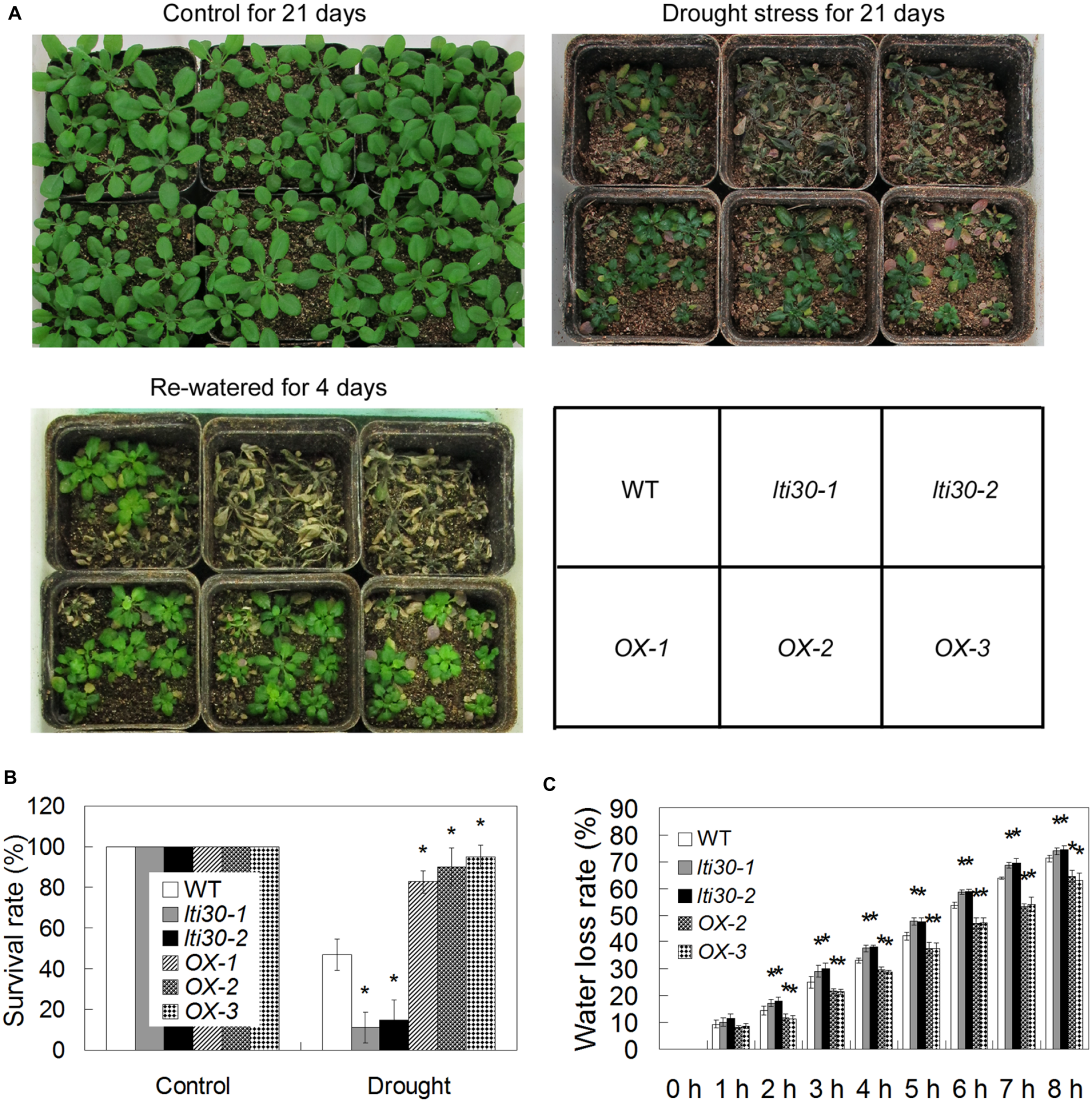
***AtLTI30* positively regulates drought stress resistance in *Arabidopsis*. (A)** The photograph showing 14-day-old *Arabidopsis* plants with well-watered for 21 days, withheld water for 21 days, and re-watered for 4 days after withheld water for 21 days. **(B)** The survival rate of different genotypes after recovery for 4 days after 21 days of drought stress treatment. **(C)** Relative leaf water loss rate *in vitro* of different lines. The results shown are the means ± SDs of three biological repeats, and asterisk symbols (^∗^) indicate the significant difference of *p* < 0.05 in comparison to WT.

To test whether *AtLTI30* regulates drought stress resistance, 14-day-old WT, *lti30* mutants, and *AtLTI30* overexpressing plants in pots were subjected to drought stress by withholding water for 21 days and then re-watering the plants for 4 days. After the drought stress treatment, the *lti30-1* and *lti30-2* mutants displayed significantly lower survival rate, while *AtLTI30* overexpressing plants exhibited higher survival rate compared with WT (**Figures 5A,B**). Consistently, the *lti30-1* and *lti30-2* mutants displayed significantly higher leave water loss rate, while *AtLTI30* overexpressing plants showed significantly lower leaf water loss rate from 2 to 8 h after detachment in comparison to WT plants (**Figure 5C**). These results indicate that *AtLTI30* positively regulates drought stress resistance.

### Modulation of *AtLTI30* Expression Affects H_2_O_2_ Accumulation

**FIGURE 6:**
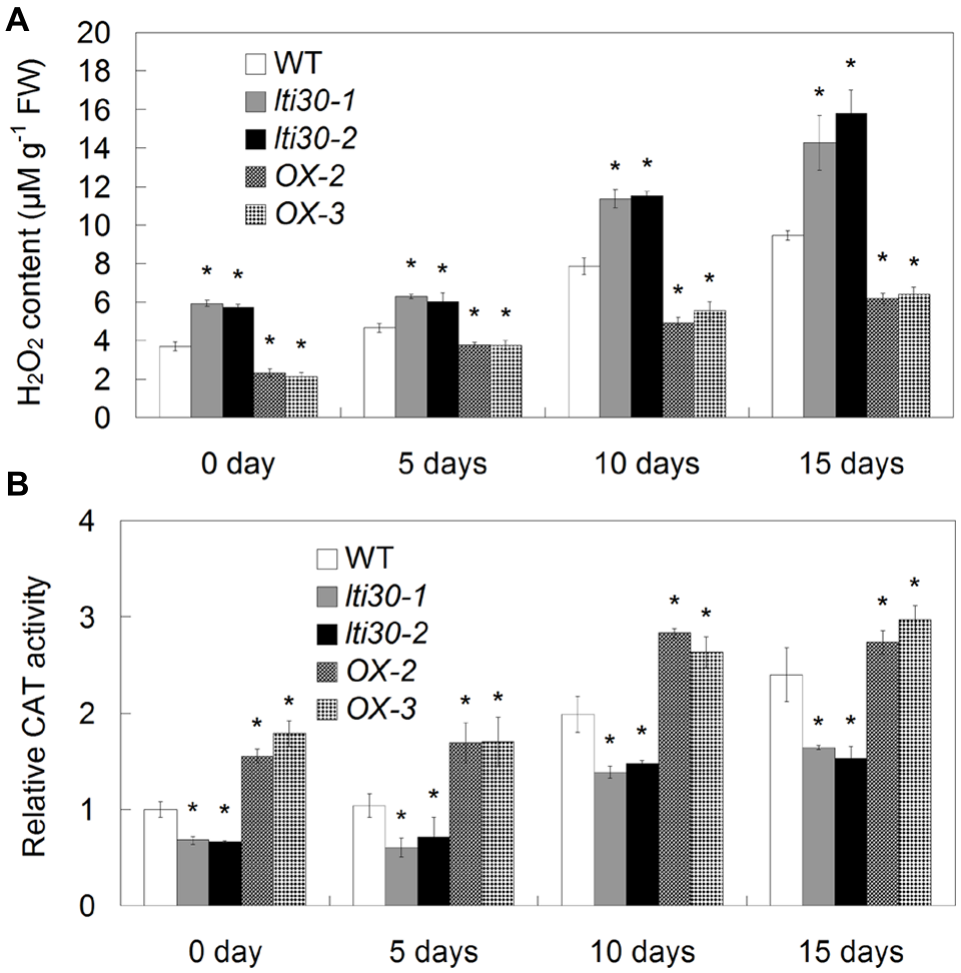
**Quantifications of H_2_O_2_ content (A) and CAT activity (B) in different lines during drought stress treatments.** For the assays, 14-d-old plant leaves were subject to drought stress by withheld water for 0, 5, 10, and 15 days. The results shown are the means ± SDs of three biological repeats, and asterisk symbols (^∗^) indicate the significant difference of *p* < 0.05 in comparison to WT.

Oxidative burst especially H_2_O_2_ accumulation occurs following drought stress in plants. We further investigated the effects of *AtLTI30* expression on H_2_O_2_ accumulation and associated antioxidant defense enzyme activity during the drought stress treatments. During the period between 0 and 15 days of drought stress, H_2_O_2_ burst was significantly displayed in WT, the *AtLTI30* knockout mutants and overexpressing plants (**Figure 6A**). In comparison to WT plants, the *AtLTI30* knockout mutants showed higher levels of H_2_O_2_ at 0, 5, 10, and 15 days of drought stress, while the *AtLTI30* overexpressing plants displayed relatively lower levels of H_2_O_2_ at these periods (**Figure 6A**).

In accordance with the H_2_O_2_ burst, the *AtLTI30* knockout mutants exhibited lower activities of AtCATs under both control and drought stress conditions, while the *AtLTI30* overexpressing plants showed relatively higher activities of AtCATs in comparison to WT (**Figure 6B**). Therefore, these results indicate that the *AtLTI30* positively regulates the activities of AtCATs, and negatively regulates H_2_O_2_ accumulation during drought stress treatment.

### *AtLTI30* Positively Regulates Drought Stress Resistance

**FIGURE 7:**
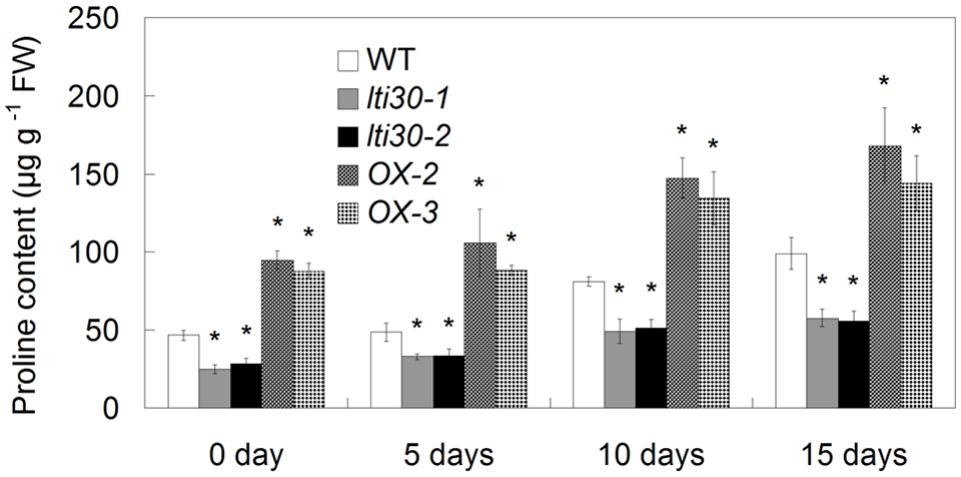
**Modulation of *AtLTI30* expression affects the endogenous proline level.** For the assays, 14-d-old plant leaves were subject to drought stress by withheld water for 0, 5, 10, and 15 days. The results shown are the means ± SDs of three biological repeats, and asterisk symbols (^∗^) indicate the significant difference of *p* < 0.05 in comparison to WT.

During the period between 0 and 15 days of drought stress, the endogenous proline content gradually increased in WT, *AtLTI30* knockout mutants and overexpressing plants (**Figure 7**). In comparison to WT plants, the *AtLTI30* knockout mutants exhibited lower proline contents at 0, 5, 10, and 15 days of drought stress, while the *AtLTI30* overexpressing plants displayed higher levels of proline at these periods (**Figure 7**).

## Discussion

As a dehydrin belonging to the group II LEA family, AtLTI30/XERO2 is widely involved in plant freezing stress resistance (Rouse et al., 1996; Nylander et al., 2001). Not only is its transcription induced under cold stress treatment, but its overexpression confers improved freezing stress resistance in *Arabidopsis* (Rouse et al., 1996; Nylander et al., 2001; Puhakainen et al., 2004). Moreover, the common expression between *CBF1/2/3* and *AtLTI30/XERO2*, and between *ABA insensitive 5* (*ABI5*) and *AtLTI30/XERO2*, indicated that *AtLTI30/XERO2* may be a direct target of both AtCBFs and AtABI5 (Chung and Parish, 2008). Using multiple combinations of mutations in the promoter of *AtLTI30/XERO2*, they also found that two of the ACGT and CRT/DRE elements are essential for both ABA and cold transcriptional induction of *AtLTI30/XERO2* (Chung and Parish, 2008). However, the possible involvement of *AtLTI30* in ABA signaling and the *in vivo* role of *AtLTI30* in plant drought stress response remains unknown.

To date, there are three effective methods to improve plant drought stress resistance. The first is to screen and identify drought tolerant varieties, and the second method is the exogenous application of multiple small molecules such as ABA, nitric oxide (NO), polyamines, hydrogen sulfide (H_2_S), and melatonin (Shi et al., 2013a, 2014a, 2015; Chan and Shi, 2015). The third method is the investigation of new genes that confer plant drought stress resistance and genetic breeding (Miao et al., 2006; Yu et al., 2008; Qin et al., 2011; Zhang et al., 2013). In this study, the new roles of *AtLTI30* in drought stress resistance may provide a useful candidate gene for drought tolerant crop genetic breeding.

In response to drought stress, plant endogenous ABA synthesis and the following ABA-responsive genes are rapidly and largely activated. Briefly, with an increase in the endogenous ABA level, ABA receptors (Pyrabactin Resistance (PYR)/PYR1-Like (PYL)/Regulatory Components of ABA Receptor (RCAR)) disrupt the interaction between type 2C protein phosphatases (PP2Cs) and sucrose non-fermenting 1 (SNF1)-related protein kinases 2 (SnRK2s) by competitively interacting with PP2Cs. Thereafter, these interaction prevent PP2Cs-mediated dephosphorylation of SnRK2s and the activation of the SnRK2s, leading to the transcriptional activation of ABA-responsive genes (Fujii et al., 2009; Cutler et al., 2010; Harb et al., 2010). Moreover, ABA also induces the accumulation of H_2_O_2_, and both ABA and ABA-induced H_2_O_2_ play important roles in plant drought stress response, especially in the modulation of stomatal closure (Zhang et al., 2001; Miao et al., 2006; Munemasa et al., 2013). In accordance with previous studies, which showed that ABA and dehydration induced the transcription level of *AtLTI30*, as evidenced by northern blot analysis (Welin et al., 1994; Rouse et al., 1996; Nylander et al., 2001; Chung and Parish, 2008), the expression of *AtLTI30* was significantly increased after ABA and drought stress treatments using *proLTI30::GUS* transgenic plants and the publicly available microarray data (http://bar.utoronto.ca/efp/cgi-bin/efpWeb.cgi) (**Figure 2**). Together with the association among ABA, H_2_O_2_ and drought stress, these results indicated the possible role of the *AtLTI30* in the process. After identifying the *AtLTI30* knockout mutants and the *AtLTI30* overexpressing plants (**Figure 3**), we found that the *AtLTI30* positively regulated plant sensitivity to ABA (**Figure 4**). In accordance with the ABA insensitive phenotype, the *lti30-1* and *lti30-2* mutants showed decreased drought stress resistance, as evidenced by a higher water loss rate and lower survival rate in comparison to WT (**Figure 5**). On the contrary, the *AtLTI30* overexpressing plants were more sensitive to ABA and exhibited improved drought stress resistance (**Figure 5**). These results suggest that *AtLTI30* may function in drought stress response in an ABA-dependent pathway. However, the stomatal response of the *AtLTI30* knockout mutants and the *AtLTI30* overexpressing plants displayed no significant difference in comparison to WT plants under mock, ABA and H_2_O_2_ conditions (Supplementary Figure S1). This result indicated that modulation of *AtLTI30* expression has no significant effect on stomatal response in *Arabidopsis*.

Reactive oxygen species (ROS) including H_2_O_2_, superoxide anions (O_2_^⋅-^), singlet oxygen (^1^O_2_) and hydroxyl radical (OH^-^) plays pivotal roles in plant drought stress responses. On one hand, H_2_O_2_ is key secondary messenger in drought stress perception and transduction (Zhang et al., 2001; Miao et al., 2006; Munemasa et al., 2013; Wang et al., 2013). On the other hand, as toxic by-products of physiological metabolism, H_2_O_2_ accumulation is rapidly and largely increased under drought stress conditions, and overproduction of H_2_O_2_ triggers serious oxidative damage and decreased drought resistance (Miller et al., 2010; Mittler et al., 2011). To cope with stress-triggered ROS overproduction and oxidative stress, plants have developed complex defense systems including both enzymatic and non-enzymatic antioxidants. Among the enzymatic enzymes, CAT catalyzes the decomposition of H_2_O_2_ into H_2_O and O_2_ and plays an essential role in controlling ROS homeostasis. In *Arabidopsis*, the *AtCATs* transcripts can be largely induced by various stress treatments including ABA, drought, salt, cold, and oxidative stresses (Du et al., 2008; Mhamdi et al., 2010; Hu et al., 2011). The interactions among nucleoside diphosphate kinase 2 (NDPK2), CAT2 or CAT3 and Salt Overly Sensitive 2 (SOS2) indicate the relationship between H_2_O_2_ and abiotic stress response (Verslues et al., 2007). Together with previous studies showing the interaction of SOS2 and other SnRK3s with ABI1 and ABI2 (Guo et al., 2002; Ohta et al., 2003) and the importance of H_2_O_2_-dependent inactivation of ABI1 and ABI2 in ABA signaling (Miao et al., 2006), CAT2 and CAT3 may occur in the same protein complex as ABI1 and ABI2, indicating the possible involvement of AtCATs in the ABA signaling pathway. In this study, the positive effect of *AtLTI30* expression on the activities of AtCATs may be directly related to drought stress-induced ROS accumulation (**Figure 6**), as well as *AtLTI30*-mediated drought stress resistance. Additionally, H_2_O_2_ is an important secondary messenger in ABA signal transduction (Miao et al., 2006; Cutler et al., 2010). Thus, the effects of *AtLTI30* expression on ABA sensitivity, H_2_O_2_ accumulation and drought resistance further suggest the dual cross-talks among these pathways.

**FIGURE 8:**
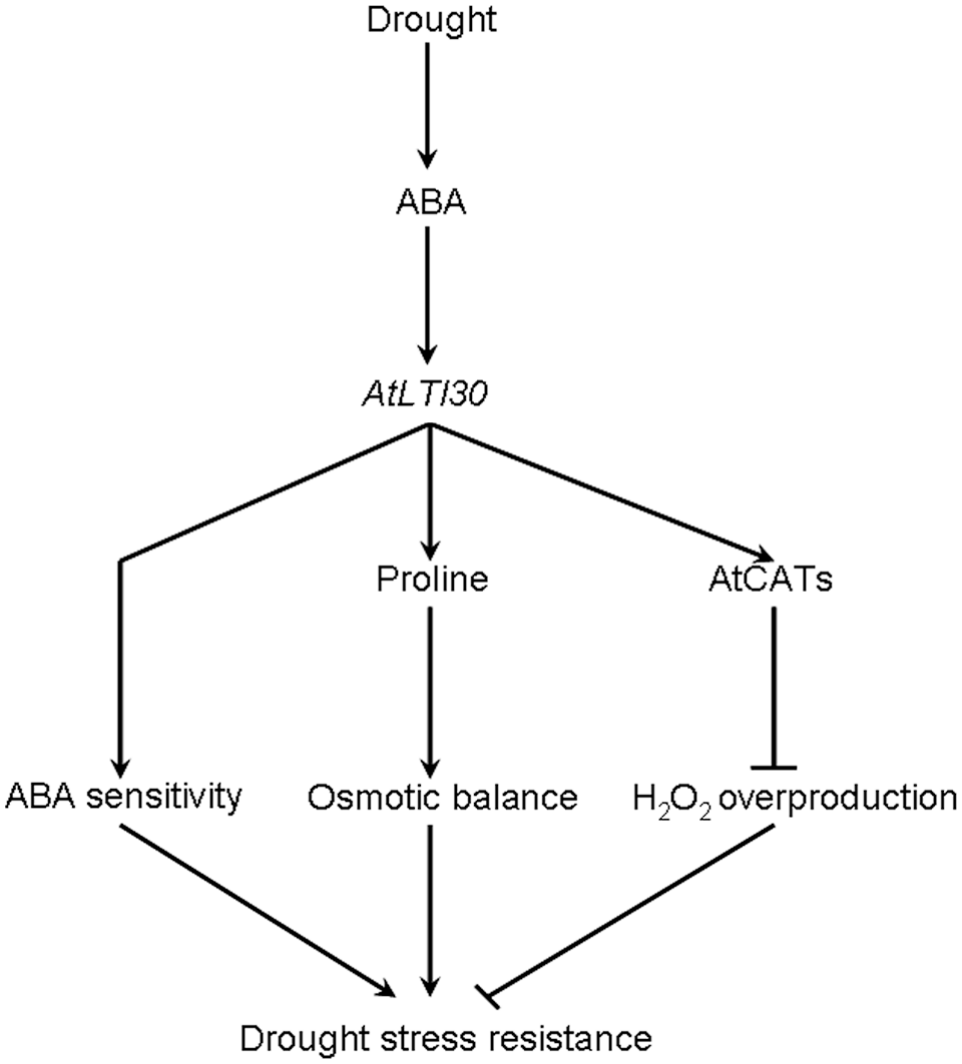
**A proposed model for *AtLTI30*-mediated drought stress response in *Arabidopsis***.

Based on these results, a model for *AtLTI30*-mediated drought stress response is proposed in this study (**Figure 8**). In response to drought stress, the endogenous ABA level is rapidly and largely induced, and thereafter induces the expression of *AtLTI30*. Firstly, overexpression of *AtLTI30* conferred ABA sensitivity, which is directly linked with ABA-mediated stress responses. Secondly, overexpression of *AtLTI30* up-regulated the activities of AtCATs, leading to less H_2_O_2_ accumulation as well as less oxidative damage under the drought stress condition. Moreover, *AtLTI30* positively regulated the endogenous level of proline, which functions as an important osmolyte in alleviating osmotic pressure under drought stress conditions (Shi et al., 2013b, 2014a), thereafter resulting in less osmotic pressure in response to drought stress. Thus, the ABA sensitivity, lower H_2_O_2_ accumulation and more proline content resulted in enhanced drought stress resistance of *AtLTI30* overexpressing plants.

Taken together, these results indicate the possible novel role of *AtLTI30* in ABA signaling, ROS accumulation and drought stress resistance. *AtLTI30* confers enhanced drought stress resistance in *Arabidopsis*, by positively regulating ABA sensitivity, CAT activity and proline accumulation, at least partially.

## Author Contributors

HS conceived and directed this study, designed and performed the experiments, analyzed the data, wrote and revised the manuscript; YC and YQ performed the experiments, analyzed the data and revised the manuscript; ZC provided suggestions and revised the manuscript. All authors approved the manuscript and the version to be published, and agreed to be accountable for all aspects of the work in ensuring that questions related to the accuracy or integrity of any part of the work are appropriately investigated and resolved.

## Conflict of Interest Statement

The authors declare that the research was conducted in the absence of any commercial or financial relationships that could be construed as a potential conflict of interest.

## References

[B1] Chan Z. Shi H. (2015). Improved abiotic stress tolerance of bermudagrass by exogenous small molecules. *Plant Signal. Behav.* 10:e991577 10.4161/15592324.2014.991577 PMC4622735 25757363

[B2] Chung S. Parish R. W. (2008). Combinatorial interactions of multiple cis-elements regulating the induction of the *Arabidopsis* XERO2 dehydrin gene by abscisic acid and cold. *Plant J.* 54 15–29. 10.1111/j.1365-313X.2007.03399.x 18088305

[B3] Clough S. J. Bent A. F. (1998). Floral dip: a simplified method for *Agrobacterium* mediated transformation of *Arabidopsis thaliana*. *Plant J.* 16 735–743. 10.1046/j.1365-313x.1998.00343.x 10069079

[B4] Cook D. Fowler S. Fiehn O. Thomashow M. F. (2004). A prominent role for the CBF cold response pathway in configuring the low-temperature metabolome of *Arabidopsis*. *Proc. Natl. Acad. Sci. U.S.A.* 101 15243–15248. 10.1073/pnas.0406069101 15383661 PMC524070

[B5] Cutler S. R. Rodriguez P. L. Finkelstein R. R. Abrams S. R. (2010). Abscisic acid: emergence of a core signaling network. *Annu. Rev. Plant Biol.* 61 651–679. 10.1146/annurev-arplant-042809-112122 20192755

[B6] Du Y. Y. Wang P. C. Chen J. Song C. P. (2008). Comprehensive functional analysis of the catalase gene family in *Arabidopsis thaliana*. *J. Integr. Plant Biol.* 50 1318–1326. 10.1111/j.1744-7909.2008.00741.x 19017119

[B7] Eriksson S. K. Kutzer M. Procek J. Gröbner G. Harryson P. (2011). Tunable membrane binding of the intrinsically disordered dehydrin Lti30, a cold-induced plant stress protein. *Plant Cell* 23 2391–2404. 10.1105/tpc.111.085183 21665998 PMC3160030

[B8] Fujii H. Chinnusamy V. Rodrigues A. Rubio S. Antoni R. Park S. Y. (2009). In vitro reconstitution of an abscisic acid signalling pathway. *Nature* 462 660–664. 10.1038/nature08599 19924127 PMC2803041

[B9] Gilmour S. J. Zarka D. G. Stockinger E. J. Salazar M. P. Houghton J. M. Thomashow M. F. (1998). Low temperature regulation of the *Arabidopsis* CBF family of AP2 transcriptional activators as an early step in cold-induced COR gene expression. *Plant J.* 16 433–442. 10.1046/j.1365-313x.1998.00310.x 9881163

[B10] Guo Y. Xiong L. M. Song C. P. Gong D. M. Halfter U. Zhu J. K. (2002). A calcium sensor and its interacting protein kinase are global regulators of abscisic acid signaling in *Arabidopsis*. *Dev. Cell* 3 233–244. 10.1016/S1534-5807(02)00229-0 12194854

[B11] Harb A. Krishnan A. Ambavaram M. M. R. Pereira A. (2010). Molecular and physiological analysis of drought stress in *Arabidopsis* reveals early responses leading to acclimation in plant growth. *Plant Physiol.* 154 1254–1271. 10.1104/pp.110.161752 20807999 PMC2971604

[B12] Hu Y. Q. Liu S. Yuan H. M. Li J. Yan D. W. Zhang J. F. (2011). Functional comparison of catalase genes in the elimination of photorespiratory H2O2 using promoter- and 3’-untranslated region exchange experiments in the *Arabidopsis* cat2 photorespiratory mutant. *Plant Cell Environ.* 33 1656–1670. 10.1111/j.1365-3040.2010.02171.x 20492555

[B13] Jefferson R. A. Kavanagh T. A. Bevan M. W. (1987). GUS fusions: beta-glucuronidase as a sensitive and versatile gene fusion marker in higher plants. *EMBO J.* 6 3901–3907. 3327686 10.1002/j.1460-2075.1987.tb02730.x PMC553867

[B14] Mhamdi A. Queval G. Chaouch S. Vanderauwera S. Breusegem F. V. Noctor G. (2010). Catalase function in plants: a focus on *Arabidopsis mutants* as stress-mimic models. *J. Exp. Bot.* 61 4197–4220. 10.1093/jxb/erq282 20876333

[B15] Miao Y. Lv D. Wang P. Wang X. C. Chen J. Miao C. (2006). An *Arabidopsis* glutathione peroxidase functions as both a redox transducer and a scavenger in abscisic acid and drought stress responses. *Plant Cell* 18 2749–2766. 10.1105/tpc.106.044230 16998070 PMC1626619

[B16] Miller G. Suzuki N. Ciftci-Yilmaz S. Mittler R. (2010). Reactive oxygen species homeostasis and signalling during drought and salinity stresses. *Plant Cell Environ.* 33 453–467. 10.1111/j.1365-3040.2009.02041.x 19712065

[B17] Mittler R. Vanderauwera S. Suzuki N. Miller G. Tognetti V. B. Vandepoele K. (2011). ROS signaling: the new wave. *Trends Plant Sci.* 16 1360–1385. 10.1016/j.tplants.2011.03.007 21482172

[B18] Mouillon J. M. Gustafsson P. Harryson P. (2006). Structural investigation of disordered stress proteins. comparison of full-length dehydrins with isolated peptides of their conserved segments. *Plant Physiol.* 141 638–650. 10.1104/pp.106.079848 16565295 PMC1475461

[B19] Munemasa S. Muroyama D. Nagahashi H. Nakamura Y. Mori I. C. Murata Y. (2013). Regulation of reactive oxygen species-mediated abscisic acid signaling in guard cells and drought tolerance by glutathione. *Front. Plant Sci.* 4:472 10.3389/fpls.2013.00472 PMC3834289 24312112

[B20] Nylander M. Svensson J. Palva E. T. Welin B. V. (2001). Stress-induced accumulation and tissue-specific localization of dehydrins in *Arabidopsis thaliana*. *Plant Mol. Biol.* 45 263–279. 10.1023/A:1006469128280 11292073

[B21] Ohta M. Guo Y. Halfter U. Zhu J. K. (2003). A novel domain in the protein kinase SOS2 mediates interaction with the protein phosphatase 2C ABI2. *Proc. Natl. Acad. Sci. U.S.A.* 100 11771–11776. 10.1073/pnas.2034853100 14504388 PMC208833

[B22] Puhakainen T. Hess M. W. Mäkelä P. Svensson J. Heino P. Palva E. T. (2004). Overexpression of multiple dehydrin genes enhances tolerance to freezing stress in *Arabidopsis*. *Plant Mol. Biol.* 54 743–753. 10.1023/B:PLAN.0000040903.66496.a4 15356392

[B23] Qin F. Shinozaki K. Yamaguchi-Shinozaki K. (2011). Achievements and challenges in understanding plant abiotic stress responses and tolerance. *Plant Cell Physiol.* 52 1569–1582. 10.1093/pcp/pcr106 21828105

[B24] Rouse D. T. Marotta R. Parish R. W. (1996). Promoter and expression studies on an *Arabidopsis thaliana* dehydrin gene. *FEBS Lett.* 381 252–256. 10.1016/0014-5793(96)00051-8 8601466

[B25] Seki M. Umezawa T. Urano K. Shinozaki K. (2007). Regulatory metabolic networks in drought stress responses. *Curr. Opin. Plant Biol.* 10 296–302. 10.1016/j.pbi.2007.04.014 17468040

[B26] Shi H. Ye T. Chen F. Cheng Z. Wang Y. Yang P. (2013a). Manipulation of arginase expression modulates abiotic stress tolerance in *Arabidopsis*: effect on arginine metabolism and ROS accumulation. *J. Exp. Bot.* 64 1367–1379. 10.1093/jxb/ers400 23378380 PMC3598423

[B27] Shi H. Ye T. Wang Y. Chan Z. (2013b). *Arabidopsis* ALTERED MERISTEM PROGRAM 1 negatively modulates plant responses to abscisic acid and dehydration stress. *Plant Physiol. Biochem.* 67 209–216. 10.1016/j.plaphy.2013.03.016 23603279

[B28] Shi H. Ye T. Han N. Bian H. Liu X. Chan Z. (2015). Hydrogen sulfide regulates abiotic stress tolerance and biotic stress resistance in *Arabidopsis*. *J. Integr. Plant Biol.* 57 628–640. 10.1111/jipb.12302 25329496

[B29] Shi H. Ye T. Zhu J. K. Chan Z. (2014a). Constitutive production of nitric oxide leads to enhanced drought stress resistance and extensive transcriptional reprogramming in *Arabidopsis.* *J. Exp. Bot.* 65 4119–4131. 10.1093/jxb/eru184 24868034 PMC4112625

[B30] Shi H. Wang X. Ye T. Chen F. Deng J. Yang P. (2014b). The Cysteine2/Histidine2-type transcription factor ZINC FINGER OF *Arabidopsis thaliana* 6 modulates biotic and abiotic stress responses by activating salicylic acid-related genes and C-REPEAT-BINDING FACTOR genes in *Arabidopsis*. *Plant Physiol.* 165 1367–1379. 10.1104/pp.114.242404 24834923 PMC4081343

[B31] Thomashow M. F. (2010). Molecular basis of plant cold acclimation: insights gained from studying the CBF cold response pathway. *Plant Physiol.* 154 571–577. 10.1104/pp.110.161794 20921187 PMC2948992

[B32] Verslues P. E. Batelli G. Grillo S. Agius F. Kim Y. S. Zhu J. (2007). Interaction of SOS2 with nucleoside diphosphate kinase 2 and catalases reveals a point of connection between salt stress and H2O2 signaling in *Arabidopsis thaliana*. *Mol. Cell. Biol.* 27 7771–7780. 10.1128/MCB.00429-07 17785451 PMC2169147

[B33] Wang P. Du Y. Zhao X. Miao Y. Song C. P. (2013). The MPK6-ERF6-ROSE7/GCC-box complex modulates oxidative gene transcription and the oxidative response in *Arabidopsis thaliana*. *Plant Physiol.* 161 1392–1408. 10.1104/pp.112.210724 23300166 PMC3585604

[B34] Welin B. V. Olson A. Nylander M. Palva E. T. (1994). Characterization and differential expression of dhn/lea/rab-like genes during cold acclimation and drought stress in *Arabidopsis thaliana*. *Plant Mol. Biol.* 26 131–144. 10.1007/BF00039526 7948863

[B35] Winter D. Vinegar B. Nahal H. Ammar R. Wilson G. V. Provart N. J. (2007). An “Electronic Fluorescent Pictograph” browser for exploring and analyzing large-scalebiological data sets. *PLoS ONE* 2:e718 10.1371/journal.pone.0000718 PMC1934936 17684564

[B36] Yang L. X. Wang R. Y. Ren F. Liu J. Cheng J. Lu Y. T. (2005). AtGLB1 enhances the tolerance of *Arabidopsis* to hydrogen peroxide stress. *Plant Cell Physiol.* 46 1309–1316. 10.1093/pcp/pci140 15930012

[B37] Yu H. Chen X. Hong Y. Y. Wang Y. Xu P. Ke S. D. (2008). Activated expression of an *Arabidopsis* HD-START protein confers drought tolerance with improved root system and reduced stomatal density. *Plant Cell* 20 1134–1151. 10.1105/tpc.108.058263 18451323 PMC2390749

[B38] Zarka D. G. Vogel J. T. Cook D. Thomashow M. F. (2003). Cold induction of *Arabidopsis* CBF genes involves multiple ICE (inducer of CBF expression) promoter elements and a cold-regulatory circuit that is desensitized by low temperature. *Plant Physiol.* 133 910–918. 10.1104/pp.103.027169 14500791 PMC219064

[B39] Zhang S. Qi Y. Liu M. Yang C. (2013). SUMO E3 ligase AtMMS21 regulates drought tolerance in *Arabidopsis thaliana*. *J. Integr. Plant Biol.* 55 83–95. 10.1111/jipb.12024 23231763

[B40] Zhang X. Zhang L. Dong F. Gao J. Galbraith D. W. Song C. P. (2001). Hydrogen peroxide is involved in abscisic acid-induced stomatal closure in *Vicia faba*. *Plant Physiol.* 126 1438–1448. 10.1104/pp.126.4.1438 11500543 PMC117144

