# Supplementary material for: Low Temperature-Induced 30 (LTI30) positively regulates drought stress resistance in Arabidopsis: effect on abscisic acid sensitivity and hydrogen peroxide accumulation
Source: Front Plant Sci. 2015 Oct 20;6:893. doi: 10.3389/fpls.2015.00893 (PMC4611175; doi:10.3389/fpls.2015.00893)
Supplement: Supplementary file 1 [file Data_Sheet_1.DOC]

**Supplemental Table S1.** The primers used for semi-quantitative RT-PCR and quantitative real-time PCR.

| **Gene** | **Locus** | **Primer** | **Sequence** |
| --- | --- | --- | --- |
| *UBQ10* | At4g05320 | UBQ10F | 5’-TCCGGATCAGCAGAGGCTTA-3’ |
|  |  | UBQ10R | 5’-TCAGAACTCTCCACCTCAAG-3’ |
| *LTI30* | At3g50970 | LTI30F | 5’-CAGAATCAAACCGGAGTGCA-3’ |
|  |  | LTI30R | 5’-AACAACGCCAGTATTACCAT-3’ |

| **Primer** | **Sequence** | **Specificity** |
| --- | --- | --- |
| LTI30OXF | 5’-TCCCCCGGGATGAATTCTCACCAGAATCA-3’ | LTI30-  pBIM |
| LTI30OXR | 5’-CCGCTCGAGCTAGTGATGACCACCGGGAA-3’ |
| pLTI30F | 5’-CGGGATCCcttttaacaccaaaaatctg-3’ | pLTI30-  pBI101.2 |
| pLTI30R | 5’-CGGGATCCttttcttctttgaatcgaaa-3’ |

**Supplemental Table S2.** The primers used for vector construction.

**
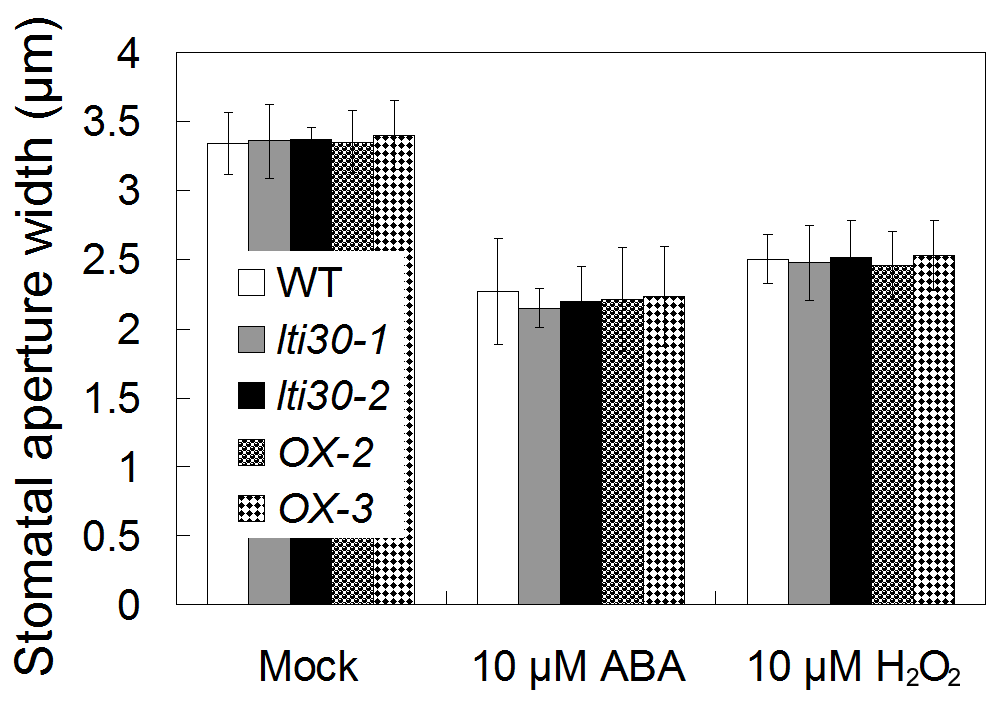
**

**Figure S1.** The stomatal responses of WT, *Atlti30* knockout mutants, and *AtLTI30* overexpressing plants to ABA and H_2_O_2_. The results shown are the means ± SDs of three biological repeats.
